# Environmental PFOA exposure and the risk of metabolic dysfunction-associated steatotic liver disease: An integrated computational toxicology and multi-omics study

**DOI:** 10.1371/journal.pone.0357970

**Published:** 2026-09-09

**Authors:** Tianyu Zhang, Yu Yuan, Chunli Lin, Chao Song, Tianrong Liao, Yuewen Sun, Hongzhen Tang

**Affiliations:** 1 Department of Acupuncture and Tuina, Guangxi University of Chinese Medicine, Nanning, Guangxi, China; 2 Ruikang Hospital Affiliated to Guangxi University of Chinese Medicine, Nanning, Guangxi, China; Sathyabama Institute of Science and Technology, INDIA

## Abstract

**Background:**

Perfluorooctanoic acid (PFOA), a pervasive environmental pollutant, has been implicated in hepatic injury and metabolic dysfunction. However, its role as an environmental risk factor in the pathogenesis of Metabolic Dysfunction-Associated Steatotic Liver Disease (MASLD) remains incompletely understood, particularly from a systems biology perspective.

**Methods:**

This study employed an integrative approach combining computational toxicology, multi-omics data analysis, and machine learning. Public databases were utilized to identify PFOA-related targets and MASLD-associated genes. A comprehensive machine learning framework comprising 113 model combinations was applied to transcriptomic datasets (GSE66676, GSE89632, GSE164760) to identify hub genes. Single-cell RNA sequencing (scRNA-seq) analysis delineated cell type-specific expression patterns. Molecular docking and dynamics simulations assessed the binding stability between PFOA and core targets, which was further validated in vitro using an FFA-induced MASLD HepG2 cell model.

**Results:**

We identified 17 shared targets between PFOA and NAFLD. Machine learning pinpointed six hub genes (NR4A2, BCL6, CASP1, SHBG, FABP4, IL10) with high diagnostic accuracy (AUC up to 0.996). scRNA-seq revealed distinct expression patterns of these genes across liver cell subtypes in MASLD. Molecular docking and dynamics simulations demonstrated stable binding of PFOA to SHBG and FABP4. In vitro experiments confirmed that PFOA exposure significantly altered the mRNA and protein expression levels of these core genes in the MASLD model.

**Conclusion:**

Our findings suggest a potential mechanistic association between PFOA exposure and MASLD pathogenesis, characterized by disruption of lipid metabolism, inflammatory responses, and immune homeostasis. While these results identify biologically plausible pathways, they do not establish epidemiological causation, and further prospective studies with quantified PFOA exposure are required to confirm causality in humans.

## Introduction

Perfluorooctanoic acid (PFOA) is a representative perfluoroalkyl substance (PFAS), widely used in industrial and consumer products due to its hydrophobic, oleophobic, surfactant-like properties, and chemical stability [1]. It is extensively employed in the manufacture of fluoropolymers, including non-stick coatings, carpets, textiles, and food packaging for stain, oil, and water resistance. PFOA is highly persistent in the environment and bioaccumulative [2], earning it the designation as a “persistent organic pollutant” [3]. It has been detected globally in water, soil, and biological specimens, including human serum and liver tissue, posing a significant public health risk [4,5]. Epidemiological studies suggest that PFOA exposure is closely associated with liver injury and lipid metabolism disorders [6], indicating it may be a potential environmental risk factor for the development and progression of metabolic dysfunction-associated steatotic liver disease (MASLD).

Non-alcoholic fatty liver disease (NAFLD) is one of the most prevalent chronic liver diseases worldwide, with an estimated global prevalence of 30.2% [7]. It is characterized by excessive lipid accumulation in hepatocytes in the absence of significant alcohol consumption or other known liver diseases [8]. The NAFLD spectrum includes simple steatosis (NAFL), non-alcoholic steatohepatitis (NASH), fibrosis, and can progress to cirrhosis and hepatocellular carcinoma, and is strongly associated with obesity, type 2 diabetes, metabolic syndrome, and aging [9].

In 2023, a multi-society Delphi consensus process led by the American Association for the Study of Liver Diseases (AASLD), the European Association for the Study of the Liver (EASL), and the Asociación Latinoamericana para el Estudio del Hígado (ALEH) proposed a new nomenclature, renaming NAFLD to MASLD [10]. In clinical practice, the two definitions identify a virtually overlapping population, with concordance rates reported between 96% and 99%, meaning that MASLD can be considered largely equivalent to NAFLD in terms of patient selection [11]. However, their diagnostic philosophies are fundamentally different: NAFLD is an exclusion-based diagnosis (requiring the ruling out of other liver diseases and significant alcohol intake), whereas MASLD is an inclusion-based diagnosis that requires the presence of hepatic steatosis plus at least one of five cardiometabolic risk factors (e.g., obesity, type 2 diabetes, hypertension, hypertriglyceridemia, or low HDL-cholesterol) [12]. This renaming carries multiple profound implications. First, it eliminates the potentially stigmatising terms “non-alcoholic” and “fatty” [13]. Second, it places metabolic dysfunction at the centre of the disease process, shifting the focus from “what it is not” to “what it is” – a positive diagnosis grounded in pathophysiology [14]. Third, the new framework introduces an overarching term, steatotic liver disease (SLD), and further stratifies the disease spectrum by adding a novel category, MetALD, for patients with metabolic dysfunction and an intermediate level of alcohol consumption [15]. Collectively, the transition from NAFLD to MASLD represents more than a mere change in acronym; it signifies a paradigm shift from a diagnosis of exclusion to one of inclusion, thereby enhancing disease awareness, improving patient-clinician communication, and enabling more precise risk stratification and management.

Current research on the risk of PFOA-induced MASLD (formerly known as NAFLD) primarily relies on epidemiological associations or isolated in vitro experiments, lacking a systems biology approach that integrates computational toxicology and multi-omics data to unravel the underlying molecular mechanisms. Therefore, this study aims to utilize public database resources and computational toxicology methods to identify potential targets of PFOA. By integrating transcriptomic data from MASLD, we applied multiple machine learning algorithms to construct high-performance diagnostic models and identify core genes. Furthermore, we analyzed the expression patterns of these core genes at the cellular subtype level using single-cell RNA sequencing (scRNA-seq). Molecular docking and molecular dynamics (MD) simulations were employed to assess the binding stability between PFOA and core targets, followed by preliminary in vitro validation. This study systematically reveals the potential molecular and cellular mechanisms by which PFOA exposure influences MASLD, providing new scientific evidence for assessing its hepatotoxicity risk and exploring potential intervention targets.

## Materials and methods

### Data collection and preprocessing

**Fig 1:**
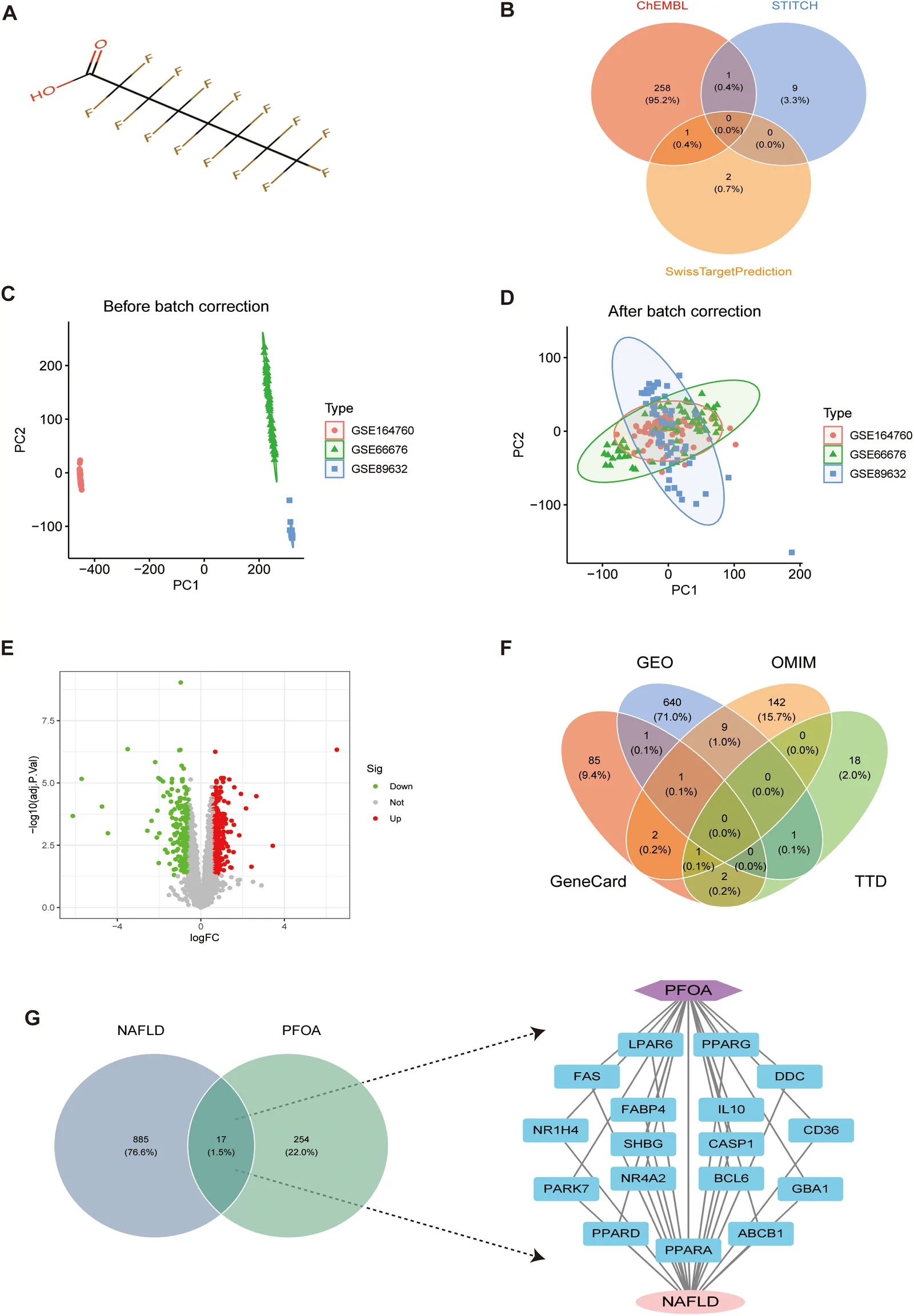
A. Two-dimensional structure of the PFOA molecule. B. Venn diagram of potential PFOA targets predicted by ChEMBL, STITCH, and SwissTargetPrediction databases. C. PCA plot of the training dataset before merging. D. PCA plot of the dataset after merging and batch correction. E. Volcano plot of differentially expressed genes (DEGs) in the NAFLD group. F. Venn diagram of overlapping disease genes from four databases (GEO, OMIM, GeneCard, TTD). G. Venn diagram of intersecting genes between PFOA and NAFLD, and the hub gene network.

The analytical workflow is detailed in Fig 1. The 3D structure and canonical SMILES (C(=O)(C(C(C(C(C(C(C(F)(F)F)(F)F)(F)F)(F)F)(F)F)(F)F)(F)F)O) of PFOA were obtained from the PubChem database. Target prediction employed a multi-strategy approach: the ChEMBL database for ligand-receptor interaction analysis; SwissTargetPrediction (probability > 0) based on chemogenomics; and STITCH (min. interaction score > 0.4). A total of 271 predicted targets, limited to the human proteome, were identified.

Nineteen NAFLD-related datasets, including 4 microarray datasets, 1 NGS dataset, and 14 scRNA-seq datasets, were downloaded from the GEO database using “NAFLD” as the search term. Details are summarized in Table 1. The microarray datasets GSE66676, GSE89632, and GSE164760 were merged to form the training set. Individual datasets underwent probe annotation and normalization before merging. The ComBat algorithm was applied to correct for batch effects in the merged training set, with efficacy verified by principal component analysis (PCA). GSE63067 and GSE135251 served as independent validation sets.

**Table 1:** Overview of datasets used in this study.

	GEO	Experiment type	Tissue	Platform	Healthy	NAFLD
Training Set	GSE66676	Array	Liver	GPL6244	34	43
	GSE89632	Array	Liver	GPL14951	24	39
	GSE164760	Array	Liver	GPL13667	6	74
Validation Set	GSE135251	High throughput sequencing	Liver	GPL18573	10	206
	GSE63067	Array	Liver	GPL570	7	11
Single-cell	GSM4041151	scRNA-seq	Liver	10X Genomics	7	–
	GSM4041154	scRNA-seq	Liver	10X Genomics		
	GSM4041156	scRNA-seq	Liver	10X Genomics		
	GSM4041159	scRNA-seq	Liver	10X Genomics		
	GSM4041160	scRNA-seq	Liver	10X Genomics		
	GSM5325534	scRNA-seq	Liver	10X Genomics		
	GSM5325535	scRNA-seq	Liver	10X Genomics		
	GSM4041162	scRNA-seq	Liver	10X Genomics	–	7
	GSM4041165	scRNA-seq	Liver	10X Genomics		
	GSM4041167	scRNA-seq	Liver	10X Genomics		
	GSM4041168	scRNA-seq	Liver	10X Genomics		
	GSM4041169	scRNA-seq	Liver	10X Genomics		
	GSM5325536	scRNA-seq	Liver	10X Genomics		
	GSM5325537	scRNA-seq	Liver	10X Genomics		

### NAFLD disease-related gene set

The merged training set was used. The limma package (v3.9.19) was used for batch effect correction and differential expression gene (DEG) analysis, with criteria |log2 (fold change)| > 0.585 and adjust P-value < 0.05, identifying 652 DEGs. A volcano plot was generated using ggplot2 (v3.0.0). Disease-related targets were screened from GeneCards, OMIM, and the Therapeutic Target Database (TTD) using “NAFLD” as the keyword. After removing duplicates, a gene library containing 902 NAFLD-related targets was constructed.

### Machine learning for core gene screening

Seventeen core genes were obtained by intersecting PFOA targets with NAFLD disease genes. To refine the 17 candidate core genes, we implemented a comprehensive machine-learning strategy utilizing 11 mainstream algorithms with specified hyperparameter configurations: regularized regression models including Lasso (λ determined by 10-fold CV), Ridge (λ by CV), and Elastic Net (Enet with α ranging from 0.1 to 0.9, λ by CV); Support Vector Machines (SVM with radial basis function kernel); Linear Discriminant Analysis (LDA); and ensemble methods including Random Forest (RF, 1000 trees, node size = 5), Gradient Boosting Machine (GBM, maximum trees = 10000, learning rate = 0.001, interaction depth = 3), Extreme Gradient Boosting (XGBoost, maximum depth = 2, learning rate = 1, nrounds determined by CV), and glmBoost (mstop determined by CV); complemented by Stepwise Logistic Regression (Stepglm with forward, backward, and both directions), Partial Least Squares regression (plsRglm), and Naïve Bayes. We employed a two-stage “feature selection + modeling” process, generating 113 unique model combinations for evaluation. All hyperparameters were optimized via 10-fold cross-validation with a fixed random seed (123) for reproducibility. Model performance was comprehensively assessed using accuracy, sensitivity, specificity, F1-score, and Area Under the Curve (AUC)-Receiver Operating Characteristic(ROC).

### SHAP model interpretability analysis

SHapley Additive explanations (SHAP) analysis was performed to interpret the decision mechanism of the best-performing model. Based on the selected key features, the kernelshap algorithm calculated the contribution value of each feature to the prediction of individual samples. Various visualizations, including feature importance bar plots, SHAP bee swarm plots, feature dependence plots, SHAP waterfall plots, and force plots for individual samples, were generated using the shapviz package.

### Protein-Protein Interaction (PPI) network construction

Core genes identified by machine learning were submitted to the GeneMANIA database to construct a PPI network, exploring functional associations and potential interactions among core targets.

### Gene Set Enrichment Analysis (GSEA)

Single-gene GSEA for the core genes was performed using the clusterProfiler package. Samples were divided into high- and low-expression groups based on the median expression of the target gene. Genome-wide expression differences between groups were calculated and ranked. Custom gene sets were used for enrichment analysis, filtering significant pathways (P < 0.05), and enrichment plots were drawn.

### Single-cell RNA sequencing data analysis

#### Data preprocessing and quality control.

scRNA-seq data (14 samples: 7 healthy, 7 NAFLD) were obtained from GEO (accessions listed in Table 1). The Seurat package (v5) was used. Cells with <200 genes detected and genes expressed in <3 cells were filtered. Quality control thresholds were: number of genes detected between 500–7500, mitochondrial gene percentage <20%, UMI count >1000, log10 (genes per UMI) > 0.8.

#### Doublet detection and filtering.

The scDblFinder package was used. Cells classified as “singlet” with a doublet score < 0.8 were retained.

#### Batch effect correction and data integration.

The Harmony algorithm integrated 14 samples. Data were normalized, 2000 highly variable genes were identified, and data were scaled regressing out mitochondrial percentage and cell cycle scores (S. Score, G2M. Score). After PCA, Harmony integration was run using the first 30 principal components and sample ID as the batch variable.

#### Cell clustering and annotation.

Based on Harmony-corrected dimensions, a K-nearest neighbor graph was constructed using the first 20 dimensions, and cells were clustered using the Louvain algorithm (resolution = 0.8). UMAP was used for visualization. Cell types were annotated based on classic marker genes.

#### Cell proportion and differential expression analysis.

Proportions of cell types per sample were calculated. Differences between healthy and NAFLD groups were compared using the Wilcoxon rank-sum test.

#### Pseudotime analysis.

Monocle2 was used for trajectory inference on macrophage and monocyte subsets. The Seurat object was converted to a cell_data_set object. After preprocessing and UMAP reduction, cells were clustered, and the trajectory graph was learned. Pseudotime values were calculated using the order_cells function.

### Molecular docking and molecular dynamics simulation

Human 3D protein structures were obtained from UniProt. The 3D structure of PFOA was obtained from PubChem. Molecular docking between PFOA and core target proteins was performed using the online CB-Dock platform to assess binding potential and interaction modes. The docking poses with the lowest Vina scores for SHBG and FABP4 with PFOA were selected as initial structures for MD simulations using Gromacs2023.2. The amber14sb force field was used for proteins, GAFF2 for PFOA, and the TIP3P water model was applied. Energy minimization was performed using the steepest descent algorithm. The system was equilibrated under NVT and NPT ensembles, followed by a 100 ns production MD simulation under constant temperature and pressure.

### Validation of NAFLD core genes

#### Reagents and materials.

HepG2 cells were obtained from the Shanghai Cell Bank of the Chinese Academy of Sciences (Shanghai, China). The cells were maintained in Dulbecco’s Modified Eagle Medium (DMEM; Gibco, USA) supplemented with 10% fetal bovine serum (FBS; Invitrogen, USA) and 100 μg/mL penicillin-streptomycin (Suzhou New Cellmei Biotechnology Co., Ltd., China).

The following primary antibodies were used for Western blot analysis: Anti-BCL6 Rabbit Polyclonal Antibody (Catalog No. DF2903; Affinity, USA), Anti-IL10 Rabbit Polyclonal Antibody (Catalog No. DF6894, Affinity, USA), Anti-SHBG Rabbit Polyclonal Antibody (Catalog No. A7450, ABclonal, China), Anti-NR4A2 Rabbit Polyclonal Antibody (Catalog No. A22011, ABclonal, China), Anti-Cleaved Caspase-1 Rabbit Polyclonal Antibody (Catalog No. A27901, ABclonal, China), Anti-FABP4 Rabbit Polyclonal Antibody (Catalog No. A25792, ABclonal, China), Anti-β-Actin Mouse Monoclonal Antibody (Catalog No. T0022, Affinity, USA), Sodium palmitate (PA) and sodium oleate (OA) were acquired as a pre-mixed solution (PA 6 mmol/L, OA 12 mmol/L, Catalog No. KC006) from Xi’an Kunchuang Technology Development Co., Ltd. (China). PFOA (purity ≥ 97%, Catalog No. B67081-100 mg) was purchased from Shanghai Yuanye Biotechnology Co., Ltd. (China). Oil Red O Stain (Catalog No. SNM345, Beijing BioLab Technologies, China). Cell Counting Kit-8 (Catalog No. C0037, Beyotime Biotechnology, China).

#### Cell culture.

HepG2 cells were cultured in complete DMEM medium containing 10% FBS and 1% penicillin-streptomycin at 37°C in a humidified atmosphere with 5% CO_2_. All experiments were conducted when cells reached approximately 80% confluence.

#### NAFLD model induction and PFOA treatment.

HepG2 cells were chosen for in vitro validation for the following reasons: HepG2 is the most widely used human hepatoma cell line for modeling MASLD-associated lipid metabolism, with well-characterized lipid accumulation pathways [16,17]; HepG2 cells have been extensively used in previous studies investigating PFOA hepatotoxicity, including assessments of lipid dysregulation and oxidative stress [18,19].

A free fatty acid (FFA) mixture was prepared by complexing sodium oleate and sodium palmitate at a 2:1 molar ratio with fatty acid-free bovine serum albumin (BSA) in serum-free DMEM. When HepG2 cells reached 70–80% confluence, the culture medium was replaced with serum-free medium containing 0.5 mM FFA-BSA complex for 24 hours to induce the NAFLD model. Control cells were treated with the same concentration of BSA without FFAs. Successful induction of intracellular lipid accumulation was confirmed by Oil Red O staining.

Following NAFLD model establishment, cells were exposed to 80 μg/mL PFOA for 24 hours [18,19]. Control and model groups were maintained in PFOA-free medium. Each experimental group included three biological replicates (n = 3).

#### Cell viability assay (CCK-8).

The effect of PFOA on the viability of HepG2 cells was evaluated using the Cell Counting Kit-8 (CCK-8) assay.HepG2 cells in the logarithmic growth phase were harvested and seeded into 96-well culture plates at a density of 20,000 cells/well, with a volume of 100 μL per well.The plates were incubated at 37°C in a humidified incubator with 5% CO2 for 24 hours to allow for complete cell adhesion.The supernatant was discarded, and fresh medium containing various concentrations of PFOA (0, 20, 40, 60, 80, and 100 μg/mL) was added. Each concentration was tested in triplicate wells.After continuous incubation for 24 hours, 10 μL of CCK-8 reagent was added to each well.The culture plates were then incubated in the dark at 37°C for 1–2 hours.The absorbance (OD value) of each well was measured at a wavelength of 450 nm using a microplate reader, and the relative cell viability of the different treatment groups was calculated.

#### Surface Plasmon Resonance (SPR) analysis.

The binding interactions between the analytes and target proteins were evaluated using a Biacore 1K instrument (Cytiva, Sweden) at 25 °C. The target proteins were immobilized onto a Series S Sensor Chip CM5 (Cytiva, Cat. No. 29149603) via standard amine-coupling chemistry using an Amine Coupling Kit (Cytiva, Cat. No. BR100050).

Briefly, after the chip surface was activated with an EDC/NHS mixture, the proteins were diluted to a concentration of 40–50 μg/mL in a 10 mM sodium acetate buffer at pH 4.0 (Cytiva, Cat. No. BR100349) and injected to achieve an immobilization level of approximately 10,000 resonance units (RU). A blank flow cell without immobilized protein was used as a reference surface to correct for non-specific binding and bulk refractive index changes.

For the binding analysis, the samples were prepared using a two-fold serial dilution in the running buffer, which consisted of PBS (137 mM NaCl, 26.8 mM KCl, 81 mM Na2HPO4, 17.6 mM KH2PO4, pH 7.2–7.4; Sangon Biotech), 0.05% Tween-20 (Sigma, Cat. No. P9416), and 5% DMSO (Sigma, Cat. No. 67-68-5). The gradient concentrations of the analytes (1.56, 3.13, 6.25, 12.5, 25, and 50 μM) were sequentially injected over the chip surface from the lowest to the highest concentration. Each cycle was performed at a constant flow rate of 30 μL/min, with an association phase of 120 s and a subsequent dissociation phase of 180 s.

All binding resonance signals were double-referenced (subtracting both the blank surface reference and the blank buffer reference). The SPR data were processed and analyzed using the Biacore Insight Evaluation Software. The binding affinities were determined by fitting the double-referenced sensograms to a 1:1 steady-state affinity model.

#### RNA extraction and quantitative real-time PCR (qRT-PCR).

Total RNA was extracted from treated cells using a Total RNA Extraction Kit. Reverse transcription was performed using the iScript cDNA Synthesis Kit. Quantitative PCR was carried out on a Bio-Rad CFX96 system using gene-specific primers (sequences provided in Table 2). The 2^(-ΔΔCt) method was applied to calculate relative mRNA expression levels, with GAPDH serving as the internal reference gene.

**Table 2:** Primer sequences for qRT-PCR.

Gene	Direction	Sequence (5’ → 3’)	Amplicon Size (bp)
**NR4A2**	Forward	GTATGGGTCCTCGCCTCAAG	191
	Reverse	AGCCTGTGCTGTAGTTGTCC	
**BCL6**	Forward	ACCGTCCATACCGGTGAGAAA	141
	Reverse	TACAAATCTGGCTCCGCAGG	
**CASP1**	Forward	CATCCCACAATGGGCTCTGT	105
	Reverse	TGAAAATCGAACCTTGCGGA	
**SHBG**	Forward	CTACTACTGCGTCACACCCG	163
	Reverse	AGGAGGAGGAGGTTTTTGTGA	
**IL10**	Forward	GGCACCCAGTCTGAGAACAG	176
	Reverse	TGGCAACCCAGGTAACCCTTA	
**FABP4**	Forward	ACAGGAAAGTCAAGAGCACCAT	152
	Reverse	AACTCTCGTGGAAGTGACGC	

#### Western blot analysis.

Cells were lysed using RIPA buffer containing protease and phosphatase inhibitors. Protein concentrations were determined with a BCA Protein Assay Kit (Beyotime Biotechnology, China). Equal amounts of protein (20–30 μg) were separated by SDS-PAGE and transferred to PVDF membranes (Millipore, USA). After blocking with 5% skim milk in TBST, membranes were incubated with primary antibodies at 4°C overnight, followed by incubation with HRP-conjugated secondary antibodies. Protein bands were visualized using an ECL chemiluminescence substrate (Millipore, USA) and imaged with a Bio-Rad ChemiDoc system. Band intensities were quantified using ImageJ software (National Institutes of Health, USA).

#### Statistical analysis.

All in vitro experiments were performed with at least three independent biological replicates (n = 3). Data are presented as mean ± standard deviation (SD). For comparisons among three or more groups (qRT-PCR, Western blot densitometry, and CCK-8 assays), one-way analysis of variance (ANOVA) was performed, followed by Tukey’s Honest Significant Difference (HSD) test for multiple pairwise comparisons. For SPR binding analysis, sensorgrams were double-referenced and fitted to a 1:1 steady-state affinity model using Biacore Insight Evaluation Software to calculate equilibrium dissociation constants (Kd). Statistical significance was defined as p < 0.05. All statistical analyses were performed using GraphPad Prism 8. 0. 2 (GraphPad Software, La Jolla, CA, USA).

### Ethical statement

This study utilized publicly available data from the Gene Expression Omnibus (GEO) database and the commercial human hepatoma cell line (HepG2), which was procured from the Shanghai Cell Bank of the Chinese Academy of Sciences (Shanghai, China). According to the national regulations of China and the policies of our institution, research involving previously generated, publicly available data sets and established commercial cell lines does not require separate ethical approval from an Institutional Review Board (IRB) or Research Ethics Committee (REC). This is because:1. The GEO data are fully anonymized and publicly accessible; 2. The HepG2 cell line is a well-established commercial resource with documented origin;3. No new human participants were involved in this study;4. No additional human tissue samples were collected. The Institutional Review Board of Ruikang Hospital, affiliated with Guangxi University of Chinese Medicine, has confirmed that this type of bioinformatics analysis using existing data and cell lines is exempt from ethical review requirements. This exemption aligns with the 1964 Helsinki Declaration and its subsequent amendments regarding the use of existing biological specimens and data.

## Results

### Identification of PFOA-NAFLD shared targets and core genes

The 2D structure of PFOA is shown in Fig 1A. Analysis of ChEMBL, STITCH, and SwissTargetPrediction databases identified 271 PFOA-related targets, with only 2 targets common across multiple databases (Fig 1B). PCA before batch correction showed clear platform-induced batch effects (Fig 1C), which were effectively removed after ComBat correction (Fig 1D). Differential expression analysis in the NAFLD training set revealed significant numbers of up- and down-regulated genes (Fig 1E). Integration of disease-associated genes from GEO (652), OMIM (155), GeneCards (92), and TTD (23) yielded 902 NAFLD-related genes (Fig 1F). Intersection with the 271 PFOA targets identified 17 core consensus genes (Fig 1G). Network analysis indicated these genes are involved in lipid metabolism (PPARG, FABP4, FAS, CD36), signaling (NR1H4, NR4A2, PARK7), inflammation regulation (IL10, CASP1, BCL6), and cytoprotection (GBA1, PARK7), forming a complex network linking PFOA exposure to NAFLD.

### Machine learning model performance and core gene selection

**Fig 2:**
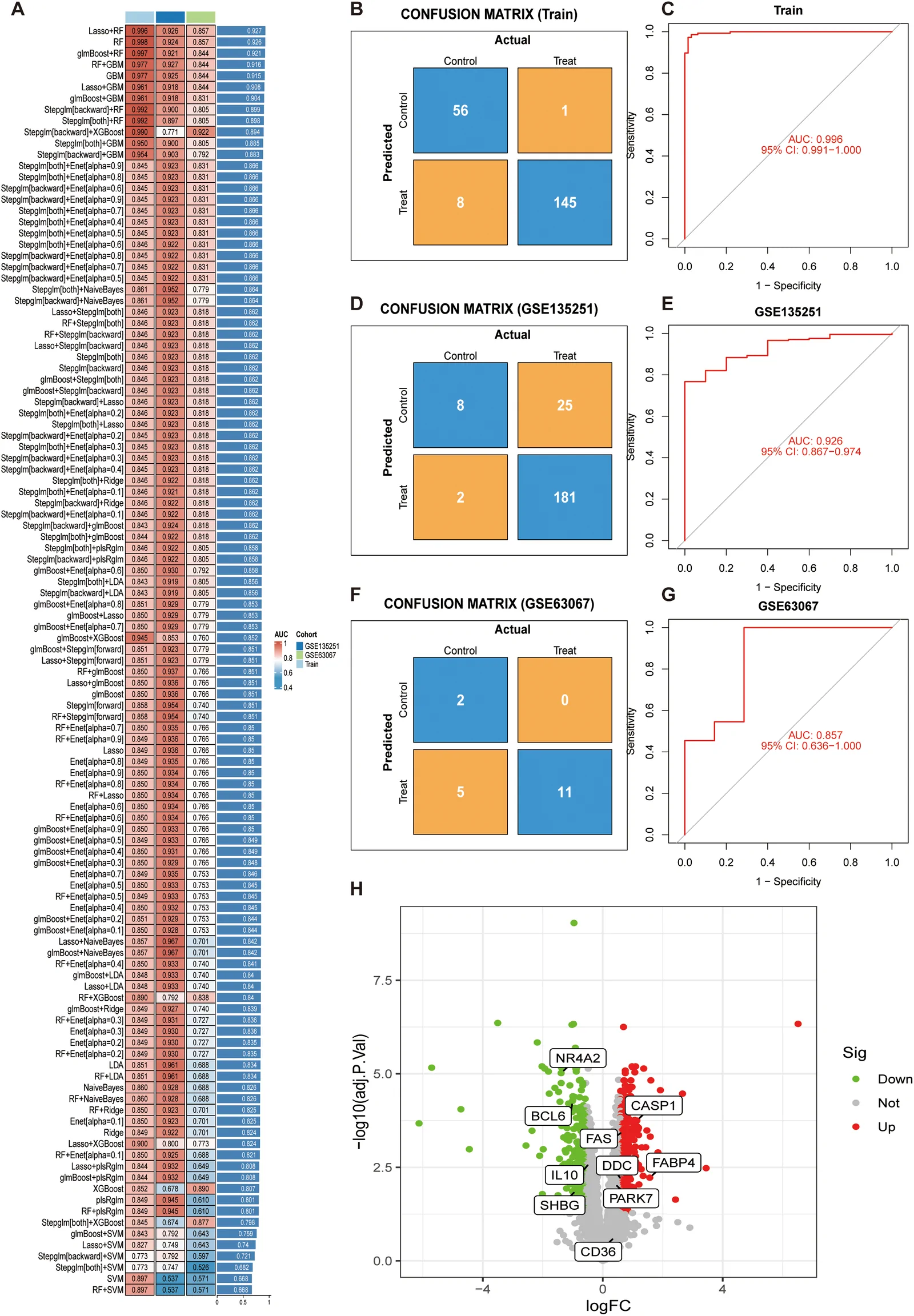
A. Heatmap comparing the AUC of 113 machine learning models across different cohorts. B-G. Confusion matrices and AUC curves for the training set and two independent validation sets (GSE135251, GSE63067), showing the model’s classification performance. H. Volcano plot of DEGs in the training set, highlighting genes selected by machine learning.

Performance evaluation of the 113 machine learning models across training and validation cohorts is shown in a heatmap of AUC values (Fig 2A). The Lasso + Random Forest (RF) algorithm combination performed best, achieving a training set AUC of 0.996 and a mean AUC of 0.927, demonstrating strong generalization capability. Confusion matrices and ROC curves for the training set and two independent validation sets (GSE135251, GSE63067) showed excellent NAFLD diagnostic performance (85% accuracy), confirming the model’s robust predictive ability (Fig 2B–2G). Differential expression analysis highlighted significant up-regulation of CASP1, DDC, FABP4, PARK7, CD36, FAS and down-regulation of BCL6, IL10, NR4A2, SHBG in NAFLD (Fig 2H). Additional model performance metrics, including precision–recall curves, decision curve analysis, and calibration curves, are shown in S2 Fig.

### Model interpretability via SHAP analysis

**Fig 3:**
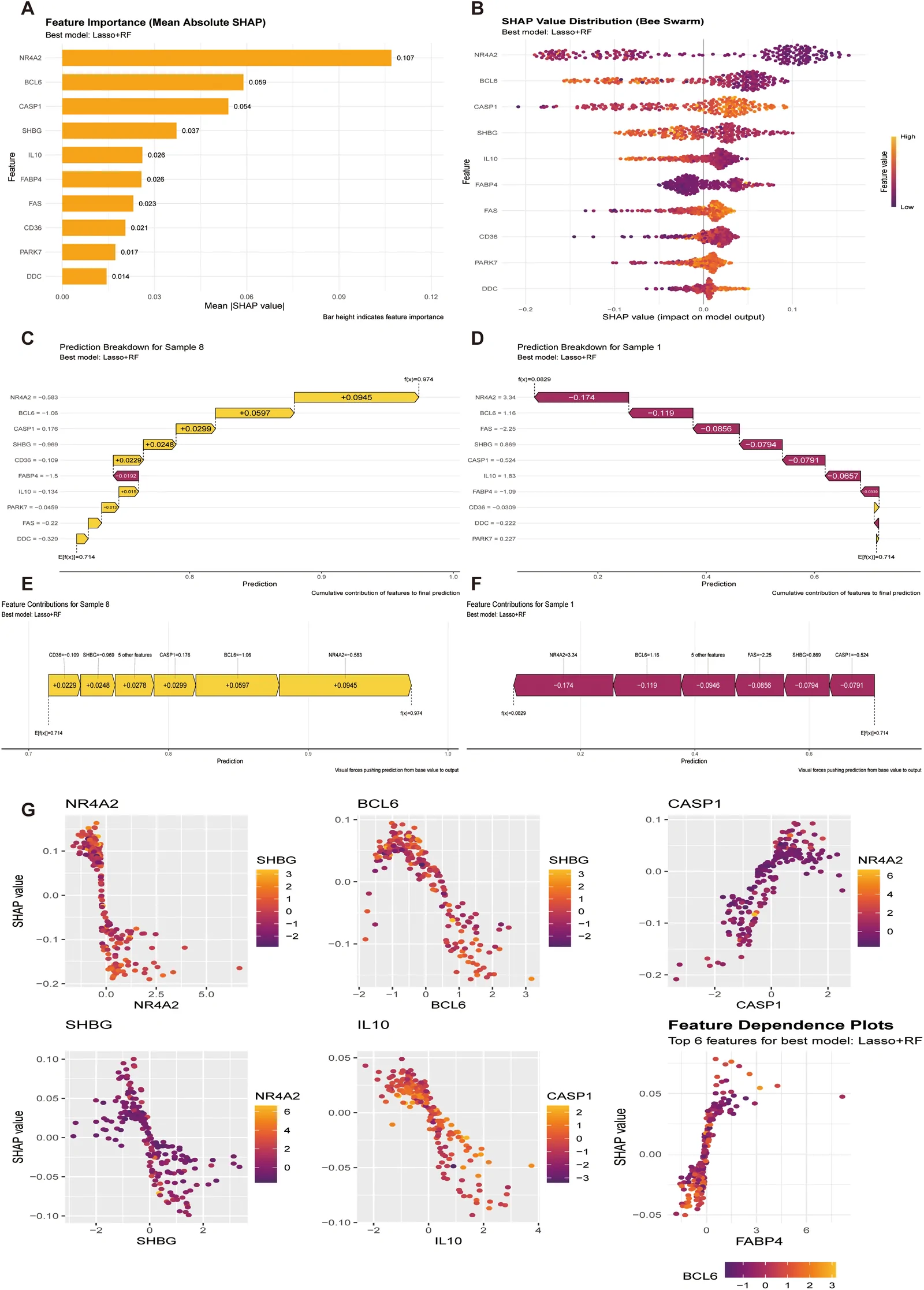
A. Bar plot of mean absolute SHAP values for feature importance. B. SHAP Bee Swarm plot. C. SHAP force plot for the prediction value of an NAFLD sample. D. SHAP force plot for the prediction value of a healthy sample. E. Bar distribution of main feature contributions for an NAFLD sample. F. Bar distribution of main feature contributions for a healthy sample. G. Scatter plots of key feature dependencies.

SHAP analysis revealed NR4A2 had the highest mean absolute SHAP value (0.107), indicating its dominant role in predictions, followed by BCL6 (0.059), CASP1 (0.054), and SHBG (0.037) (Fig 3A). The Bee Swarm plot showed wide distributions of SHAP values for these features, indicating high discriminatory power (Fig 3B). Decomposition plots for NAFLD and healthy samples illustrated how individual features pushed the model output toward the respective class prediction, with NR4A2 and BCL6 being primary drivers (Fig 3C–3F). Dependence plots (Fig 3G) suggested potential interactions, such as a negative correlation between NR4A2 and SHBG SHAP values, and a positive correlation between IL10 and CASP1 SHAP values.

### Functional characterization of core genes

**Fig 4:**
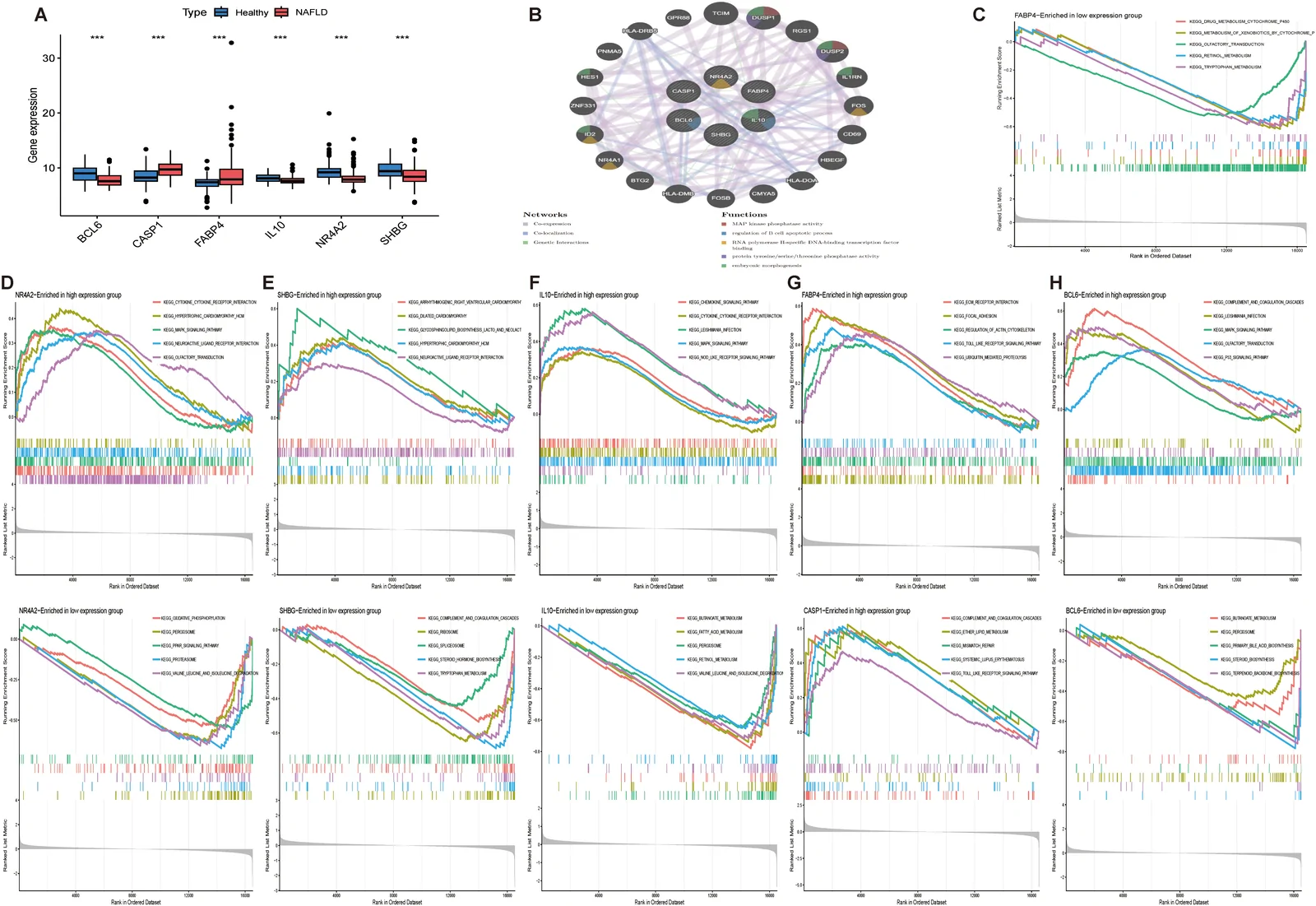
A. Box plots of key gene expression (***P < 0.001). B. Protein-protein interaction (PPI) network of key genes and their functional annotations. C-H. GSEA enrichment curves for key genes (CASP1, FABP4, BCL6, IL10, NR4A2, SHBG), displaying major enriched pathways in high- and low-expression groups.

The top six SHAP genes were defined as final core genes. Box plots confirmed significant expression differences (P < 0.001): BCL6, IL10, NR4A2, SHBG were down-regulated, while CASP1 and FABP4 were up-regulated in NAFLD (Fig 4A). The PPI network (Fig 4B) implicated these genes in functions like protein tyrosine/serine/threonine phosphatase activity, RNA polymerase II-specific DNA-binding transcription factor activity, regulation of B-cell apoptosis, and MAP kinase phosphatase activity. GSEA revealed pathway associations: CASP1 high-expression enriched in complement/coagulation cascades and Toll-like receptor signaling (Fig 4C); NR4A2 low-expression enriched in oxidative phosphorylation and PPAR signaling (Fig 4D); SHBG low-expression enriched in complement/coagulation and steroid biosynthesis (Fig 4E); IL10 low-expression enriched in fatty acid metabolism (Fig 4F); FABP4 high-expression enriched in ECM-receptor interaction and focal adhesion (Fig 4G); BCL6 low-expression enriched in peroxisome and bile acid biosynthesis (Fig 4H). This indicates a coordinated network involving metabolism, immunity, and cell signaling in NAFLD pathogenesis.

### Single-cell transcriptomic landscape and core gene expression

**Fig 5:**
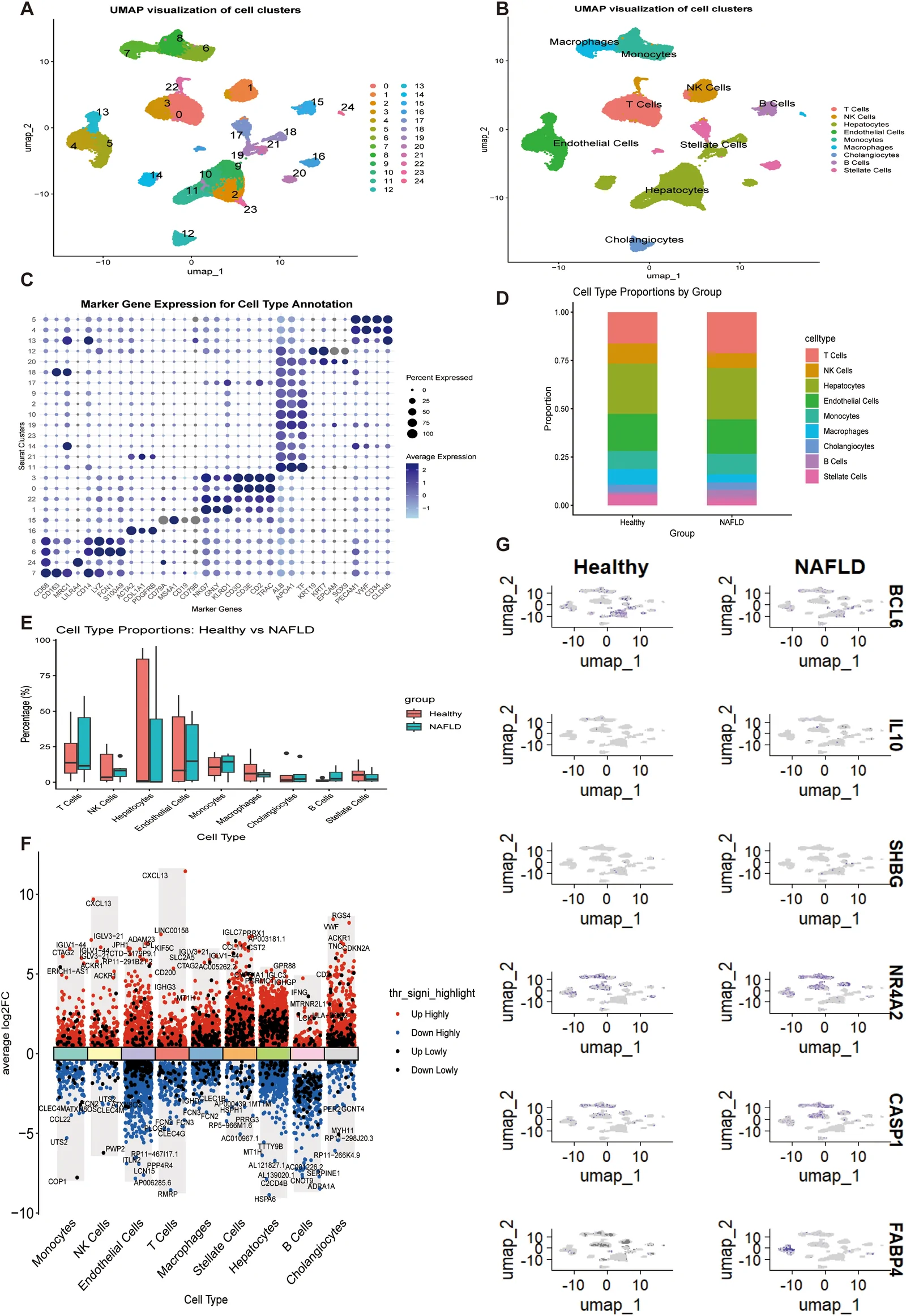
A. UMAP plot showing the clustering distribution of all cells. B. UMAP clustering and annotation of major cell types. C. Expression level and distribution of marker genes for different cell subpopulations (DotPlot). D-E. Comparison of the proportions of major cell subpopulations between NAFLD and healthy groups. F. Distribution of differentially expressed genes across different cell subpopulations (grouped by average log2FC). G. Localized visualization of key gene expression in UMAP space across cell subpopulations.

UMAP visualization identified 24 distinct cell clusters (Fig 5A), annotated into major liver cell types (Fig 5B). Dot plot analysis confirmed the expression of canonical cell type markers (Fig 5C). Proportion analysis revealed significant shifts in the NAFLD liver immune microenvironment, including increased T cell proportion and decreased NK cell, hepatocyte, and macrophage proportions (Fig 5D and 5E). Differential expression analysis across cell types showed distinct patterns of up- (red) and down-regulated (blue) genes in NAFLD (Fig 5F). Spatial expression visualization of the six core genes on the UMAP plot revealed cell type-specific localization (Fig 5G). For instance, BCL6 expression decreased in NAFLD across endothelial, NK, and hepatocyte clusters; CASP1 and FABP4 increased in neutrophils/macrophages/T cells and endothelial cells, respectively; NR4A2 was prominent in immune cells; IL10 and SHBG were primarily expressed in hepatocytes.

### Cellular trajectory analysis

**Fig 6:**
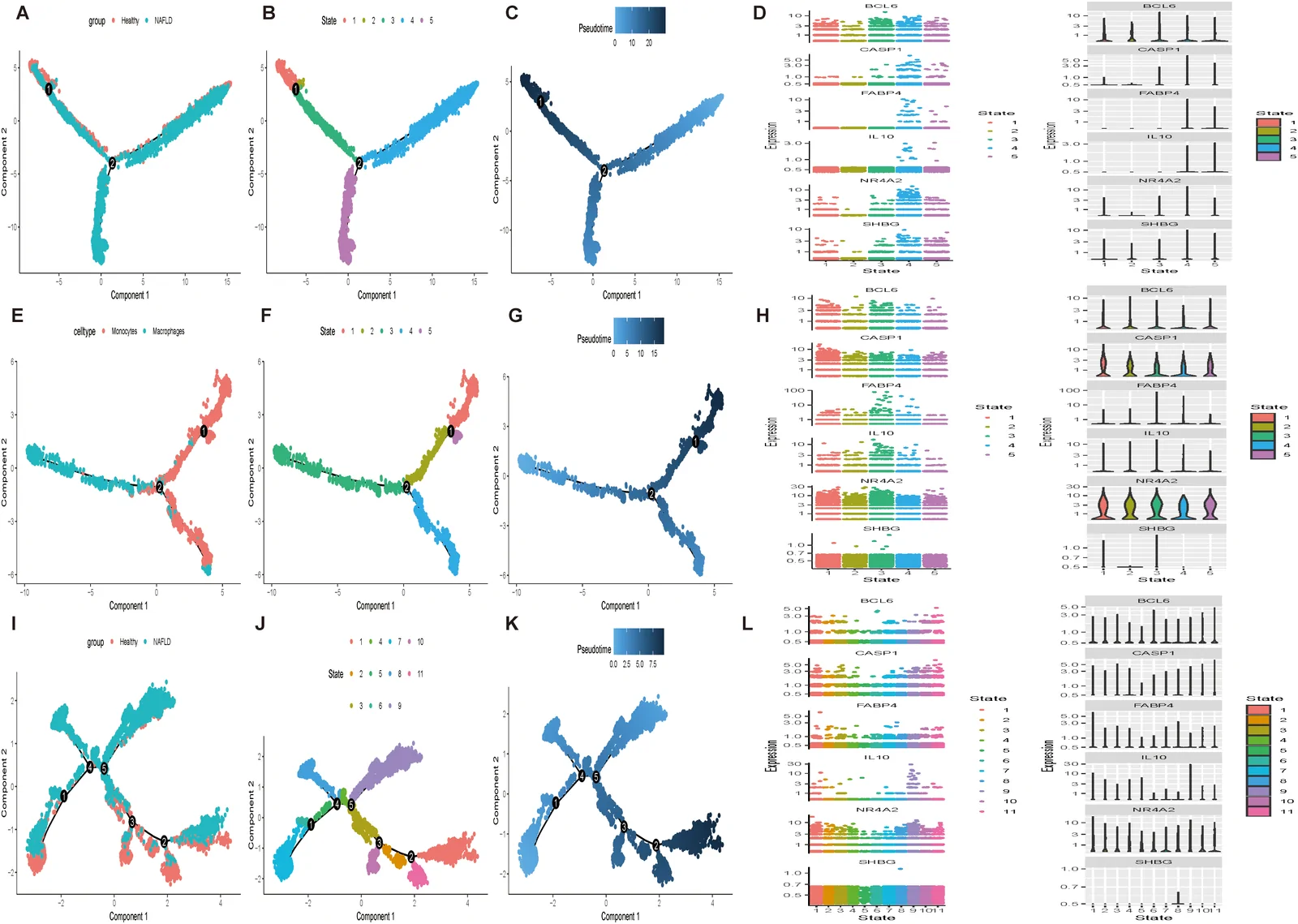
A. Dimensionality reduction distribution of hepatocytes in healthy vs. NAFLD groups. B. Dimensionality reduction distribution of classified hepatocyte states. C. Pseudotime trajectory distribution of hepatocytes. D. Dot plot and expression distribution of marker genes across hepatocyte stages. E. Dimensionality reduction distribution of monocyte and macrophage groups. F. Dimensionality reduction distribution of classified monocyte and macrophage states. G. Pseudotime trajectory of monocyte and macrophage distribution. H. Dot plot and expression distribution of marker genes for different states of monocytes and macrophages. I. Dimensionality reduction distribution of T cells in healthy vs. NAFLD groups. J. Dimensionality reduction distribution of classified T cell states. K. Pseudotime trajectory distribution of T cells. L. Dot plot and expression distribution of marker genes for various T cell states.

Analysis of hepatocytes (Fig 6A–6D), monocyte-macrophages (Fig 6E–6H), and T cells (Fig 6I–6L) revealed distinct clustering and state transitions between health and NAFLD. Pseudotime analysis reconstructed differentiation trajectories. Expression of the core genes (BCL6, CASP1, FABP4, IL10, NR4A2, SHBG) varied dynamically across cell states and along pseudotime, suggesting their involvement in cell state transitions during MASLD progression.

### Molecular docking and dynamics simulations

**Fig 7:**
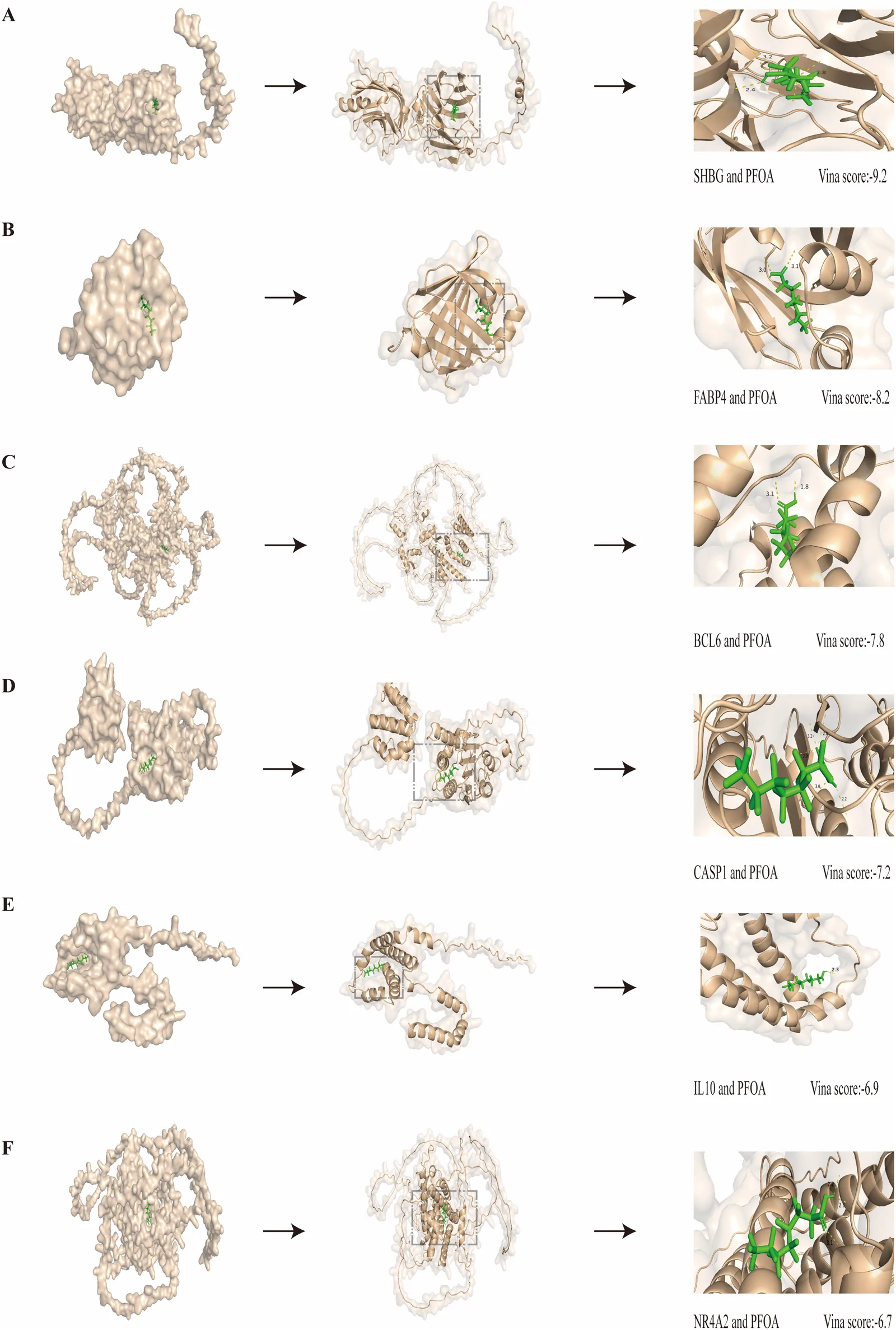
A-F. Molecular docking results of PFOA with SHBG, FABP4, BCL6, CASP1, IL10, and NR4A2.

**Fig 8:**
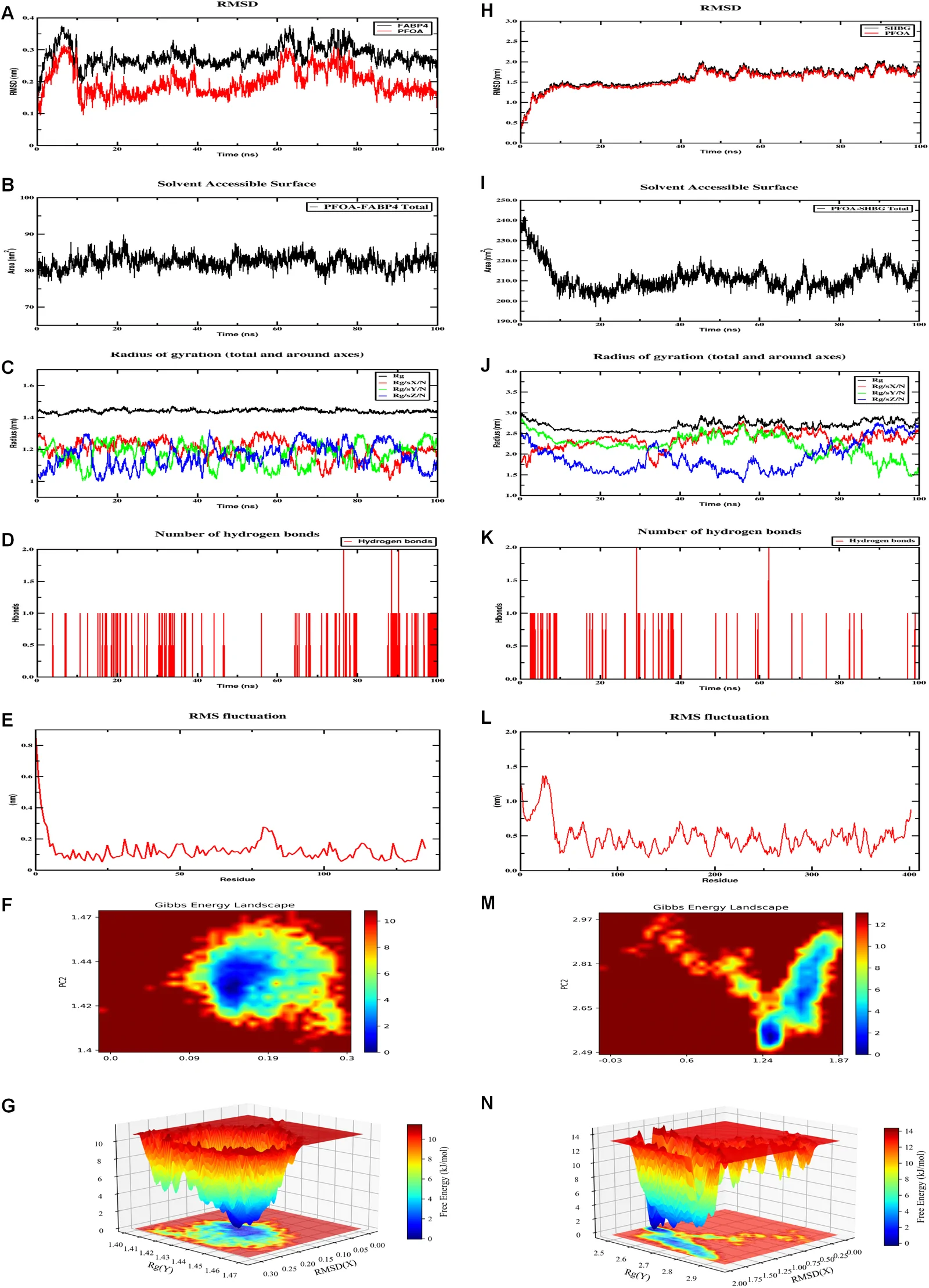
A-G. Molecular dynamics simulation results for the FABP4-PFOA complex. H-N. Molecular dynamics simulation results for the SHBG-PFOA complex.

Molecular docking revealed favorable binding between PFOA and all six core proteins (Fig 7A–7F). Vina scores were: BCL6: −7.8; CASP1: −7.2; FABP4: −8.2; IL10: −6.9; NR4A2: −6.7; SHBG: −9.2, with FABP4 and SHBG showing the strongest binding potential. MD simulations for FABP4-PFOA (Fig 8A–8G) and SHBG-PFOA (Fig 8H–8N) complexes over 100 ns showed stable RMSD, SASA, radius of gyration, and hydrogen bonding profiles, indicating structural stability and robust binding. RMSF analyzes identified flexible regions potentially involved in binding. Free energy landscapes confirmed the stability of both complexes.

### In vitro experimental validation

**Fig 9:**
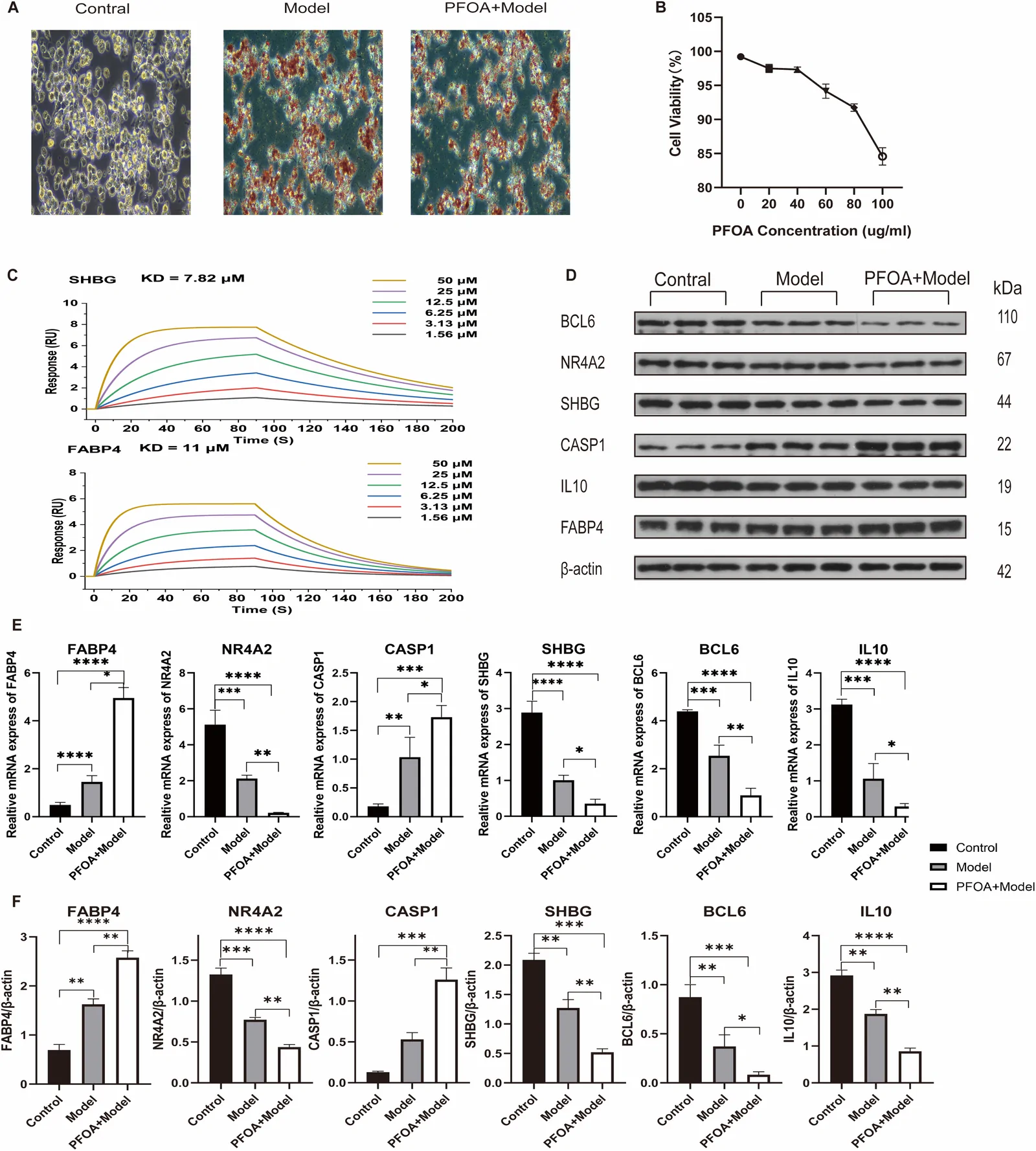
A. Oil Red O cell staining. B. Effect of PFOA on cell viability (CCK-8 assay). C. Kinetic analysis of SHBG and FABP4 interactions (SPR sensorgrams). D.Western Blot (WB) bands. E. Bar chart of Q-PCR results (*p < 0.05, **p < 0.01, ***p < 0.001, ****p < 0.0001). F. Bar chart of WB grayscale analysis (*p < 0.05, **p < 0.01, ***p < 0.001, ****p < 0.0001).

To evaluate the potential toxicity of PFOA in vitro, we first examined the effect of varying concentrations of PFOA exposure on the viability of HepG2 cells using the CCK-8 assay. The results indicated that PFOA treatment within the concentration range of 0–100 μg/mL did not induce significant cell death. Even at the highest tested concentration of 80 μg/mL, the relative cell viability remained at approximately 90%(Fig 9B). These findings demonstrate that PFOA exhibits extremely low acute cytotoxicity within this concentration range. Having confirmed that PFOA possesses a broad safe concentration window, we further investigated its potential molecular targets by evaluating the direct interaction between PFOA and two key proteins—lipid metabolism-related protein FABP4 and sex hormone-binding globulin (SHBG)—using Surface Plasmon Resonance (SPR) technology. Kinetic fitting results confirmed that PFOA specifically binds to both proteins, with equilibrium dissociation constants of 11 μM for FABP4 and 7.82 μM for SHBG(Fig 9C). Oil Red O staining confirmed successful induction of lipid accumulation in the FFA-treated NAFLD cell model (Fig 9A). qRT-PCR (Fig 9E) and Western Blot (Fig 9D and 9F) analyzes confirmed that PFOA exposure significantly altered the mRNA and protein expression levels of the core genes (NR4A2, BCL6, CASP1, SHBG, FABP4, IL10) in the NAFLD model, consistent with the bioinformatics predictions.The original Western blot images are provided in S1 Fig.

## Discussion

Against the backdrop of escalating environmental pollution and its documented links to digestive pathologies, this study investigates the potential role of PFOA in MASLD. The pervasive and persistent nature of PFOA, a recalcitrant pollutant, coincides with the rising global incidence of MASLD, warranting focused research into this specific exposure-disease relationship.

This study integrated computational and experimental approaches to systematically investigate the molecular link between PFOA exposure and MASLD risk. We identified six core genes (NR4A2, BCL6, CASP1, SHBG, FABP4, IL10) central to this interaction, functioning in lipid metabolism, inflammation, and immune regulation.

BCL6 functions as a critical transcriptional repressor [20]. A reduction in its hepatic expression is considered a key mechanism contributing to excessive lipid deposition in hepatocytes. BCL6 directly suppresses the gene expression of the fatty acid transporter CD36, thereby limiting the uptake of free fatty acids into hepatocytes [21]. Under MASLD conditions, decreased BCL6 levels relieve this transcriptional repression, leading to aberrantly increased hepatic fatty acid uptake and exacerbating hepatocellular lipid accumulation, i.e., hepatic steatosis. Studies have shown that BCL6 deletion significantly enlarges inguinal adipocytes and increases adipose tissue mass in rats [22]. Furthermore, BCL6 has been identified as a sex-dependent, growth hormone-regulated hepatic transcription factor that drives increased susceptibility to high-fat diet-induced MASLD in male mice [23].

IL-10, a central anti-inflammatory cytokine, plays a key role in inflammatory regulation. Its deficiency is a crucial factor in the transition from MASLD to NASH. Substantial evidence indicates that IL-10 levels are significantly reduced in MASLD [24–26]. IL-10 may exert its effects by modulating the MyD88–Toll-like receptor–NF-κB signaling pathway [27]. Previous studies have demonstrated that IL-10 acts as a negative regulator of inflammation, inhibiting T cells from secreting multiple pro-inflammatory cytokines such as IL-2, IL-6, IL-13, and IL-14 [28]. It also effectively suppresses the overactivation of immune cells including macrophages in the liver [29] and antagonizes the effects of pro-inflammatory factors like TNF-α [30]. The observed low serum IL-10 levels in MASLD patients indicate impaired endogenous anti-inflammatory capacity. This defect prevents the effective containment of inflammation triggered by various damage signals, creating an uncontrolled inflammatory microenvironment that ultimately drives the progression from hepatic inflammation and necrosis to fibrosis.

SHBG is best known for its role as a carrier protein that transports sex steroids in the blood and regulates their bioavailability and access to target tissues and cells [31]. Low SHBG levels are not only a sensitive biomarker for MASLD but may also directly contribute to disease pathogenesis [32,33]. SHBG overexpression has been shown to reduce PPARγ levels via activation of the ERK-1/2 MAPK pathway, thereby downregulating key lipogenic enzymes and hepatic triglyceride content, ultimately attenuating hepatic steatosis [34]. Conversely, SHBG downregulation, mediated by hepatic lipid accumulation and increased pro-inflammatory cytokines as observed in obesity and type 2 diabetes, may accelerate MASLD progression, creating a vicious cycle that further reduces SHBG production. The relationship between SHBG and metabolic disorders can also be explained through androgen dynamics. Lower SHBG typically leads to increased circulating levels of free bioactive androgens, which may disrupt insulin secretion and pancreatic β-cell function, thereby exacerbating metabolic syndrome and insulin resistance [35]. Additionally, reduced SHBG expression in MASLD may be secondary to inflammation; TNF-α, via activation of JNK and NF-κB, has been shown to decrease SHBG production in HepG2 cells [36].

NR4A2, a member of the NR4A subfamily of nuclear receptors, plays vital roles in regulating cell growth, metabolism, inflammation, and other biological functions [37]. It has been demonstrated that NR4A2 inhibits the activation and proliferation of hepatic stellate cells—a central event in liver fibrogenesis [38]. The significant decrease in NR4A2 expression observed in the livers of NAFLD/MASLD patients likely weakens the body’s intrinsic anti-fibrotic capacity, thereby creating conditions conducive to the progression of liver fibrosis and cirrhosis [39]. Furthermore, studies confirm that NR4A2 binds to the promoter region of OTUD7B and positively transcriptionally regulates its expression. OTUD7B overexpression ameliorates hepatic inflammation and steatosis in MASLD, whereas downregulation of NR4A2 exacerbates liver inflammation, injury, and fibrosis progression [40].

CASP1 serves as the pivotal effector protein in the inflammasome signaling pathway and acts as an “accelerator” in NASH pathogenesis. In the lipotoxic environment of MASLD, danger signals such as excess free fatty acids and cholesterol crystals activate the NLRP3 inflammasome, which in turn cleaves and activates CASP1 [41]. Activated CASP1 induces liver injury through two major pathways: first, it catalyzes the maturation of pro-inflammatory cytokine precursors pro-IL-1β and pro-IL-18 into their highly active forms, triggering a potent inflammatory cascade [42]; second, it cleaves GSDMD protein, inducing pyroptosis—a form of programmed cell death accompanied by the release of abundant inflammatory mediators—further amplifying intrahepatic inflammation and injury [43]. Some studies also suggest that CASP1 can modulate the composition and diversity of the gut microbiota, thereby influencing hepatic lipid composition and metabolism and promoting MASLD progression [44]. Research has confirmed that using a CASP1 inhibitor can alleviate the progression of NASH, liver fibrosis, and insulin resistance [45]. Consequently, targeted inhibition of CASP1 has become a strategy of significant interest in MASLD therapeutics.

Fatty Acid Binding Protein 4 (FABP4), a member of the lipid-binding protein family, actively facilitates the transport of fatty acids to various cellular organelles [46]. FABP4 acts as a “messenger” linking adipose tissue dysfunction to liver injury, bridging systemic metabolic disturbances and local hepatic inflammation. In states of obesity and insulin resistance, the release of FABP4 from adipose tissue into the circulation increases [47]. Elevated circulating FABP4 promotes lipolysis, thereby increasing the flux of fatty acids to the liver [48]. On the other hand, it can act directly on the liver to promote hepatic lipid deposition, insulin resistance, and the activation of inflammatory signaling pathways [49]. FABP4 also plays a significant role in macrophage inflammatory activation [50]. Studies have shown that specific ablation of FABP4 in macrophages is sufficient to protect mice from diet-induced dyslipidemia [51]. Clinical studies further confirm that serum FABP4 levels positively correlate with the severity of MASLD and the stage of fibrosis, underscoring its dual value as both a pathogenic factor and a disease activity marker [52].

These molecules form an interconnected network: Metabolic dysregulation (low SHBG, high FABP4, low BCL6) initiates steatosis. Inflammation amplification follows, driven by CASP1 activation, with failed resolution due to low IL10 and BCL6, leading to NASH. In the fibrotic phase, low NR4A2 fails to restrain stellate cell activation, promoting fibrosis.

Our computational and in vitro findings identify statistical associations and mechanistic perturbations rather than epidemiological causation. The human transcriptomic data are cross-sectional and lack quantified PFOA exposure measurements; therefore, they cannot establish a temporal or causal relationship between PFOA exposure and NAFLD development in human populations. Instead, our study employs a systems toxicology framework that integrates multi-omics correlations, machine learning predictions, and experimental validation to generate mechanistic hypotheses. The direct binding of PFOA to FABP4 and SHBG (confirmed by SPR), along with the modulation of these genes in a cellular NAFLD model, provides in vitro evidence of biological plausibility. However, definitive causal inference would require prospective cohort studies with quantified PFOA exposure, dose-response assessments, and controlled animal models. We have therefore framed our conclusions as identifying potential molecular mechanisms that warrant further investigation, rather than establishing PFOA as a direct cause of NAFLD in humans.

## Conclusion

In conclusion, this study establishes a framework linking PFOA exposure to MASLD risk, proposing a mechanism involving the dysregulation of a core gene network (NR4A2, BCL6, CASP1, SHBG, FABP4, IL10) within specific liver cell populations, disrupting metabolic and immune homeostasis. These findings enhance our understanding of environmental hepatotoxicity and identify potential biomarkers and therapeutic targets for MASLD.

## Study limitations and future directions

This study has limitations. The merged GEO datasets have heterogeneity; despite ComBat correction, platform differences, sample size variations, and disease stages may cause bias. For example, GSE164760 has 6 healthy controls vs. 74 NAFLD samples, skewing the model. Lack of clinical covariates (e.g., fibrosis stage, NAS score, BMI, sex) prevents sub-group analyses and may confound gene-NAFLD associations, such as for SHBG. However, evidence supports six core genes: (1) consistent selection across 113 model combinations; (2) high SHAP importance; (3) validation on two independent cohorts; (4) single-cell confirmation; (5) in vitro validation of mRNA and protein changes. Future prospective studies with stratified recruitment are needed to confirm generalizability. In silico PFOA target predictions may have false positives; the initial set of 271 proteins from databases may include biases, but filtering reduces risk. Four core targets (NR4A2, BCL6, CASP1, IL10) lack SPR binding confirmation; future studies with ITC, CETSA, or co-crystallization are required. The study is exploratory; cross-sectional data lack PFOA exposure measures, preventing causal inference. In vitro findings may not translate to humans. Causal relationships require prospective studies with exposure biomarkers and confounder adjustment. HepG2 cells have limitations as hepatoblastoma-derived; they may not mimic primary hepatocytes, especially for SHBG regulation. SHBG data should be cautious. Future studies with primary cells or organoids are needed. While our computational predictions were supported by SPR validation for FABP4 and SHBG, the binding affinities and biological relevance of PFOA interactions with the remaining four core targets (NR4A2, BCL6, CASP1, IL10) require further experimental confirmation using additional biophysical techniques such as isothermal titration calorimetry (ITC) or fluorescence polarization assays. The computational predictions for these targets should therefore be interpreted as hypotheses requiring experimental testing rather than established interactions. Environmental relevance of PFOA concentration used. The 80 μg/mL PFOA concentration in vitro is higher than general population serum levels. This discrepancy stems from differences between acute in vitro and chronic in vivo exposure: cultured cells lack physiological clearance mechanisms (renal excretion, hepatobiliary elimination) and protein-binding dynamics that maintain low steady-state concentrations in humans. CCK-8 assay confirmed 80 μg/mL is non-cytotoxic (>90% viability), ensuring gene expression changes reflect specific molecular perturbations, not general cytotoxicity. This concentration aligns with previous PFOA hepatotoxicity studies and was chosen to detect gene expression changes within 24 hours. Thus, in vitro findings represent biological plausibility and mechanistic potential, not direct quantitative predictions at environmental exposure levels. Future studies should employ concentration-response designs with lower, physiologically relevant doses and chronic exposure models to bridge this translational gap. Finally, this study focused solely on PFOA, whereas real-world exposure involves mixtures of pollutants, warranting future research into combined toxicological effects.

## Supporting information

S1 Fig Original Western blot images of target proteins. Raw Western blot bands of FABP4, cleaved caspase-1, BCL6, SHBG, NR4A2, IL-10, and β-actin in control, FFA-induced NAFLD model, and PFOA-treated model groups of HepG2 cells.
(TIF)

S2 Fig Performance evaluation of the optimal machine learning model across cohorts. (A–C) Precision–recall curves; (D–F) decision curve analysis; (G–I) calibration curves for the training set and two independent validation sets.
(TIFF)

S1 File Graphical abstract. (PNG)
